# Head and Neck Osteosarcoma—The Ongoing Challenge about Reconstruction and Dental Rehabilitation

**DOI:** 10.3390/cancers12071948

**Published:** 2020-07-18

**Authors:** Andrea Cassoni, Edoardo Brauner, Resi Pucci, Valentina Terenzi, Nicolò Mangini, Andrea Battisti, Marco Della Monaca, Alessandro Ciolfi, Federico Laudoni, Stefano Di Carlo, Valentino Valentini

**Affiliations:** 1 Department of Oral and Maxillofacial Sciences, Sapienza University of Rome; Via Caserta 6, 00161 Rome, Italy; andrea.cassoni@uniroma1.it (A.C.); edoardo.brauner@uniroma1.it (E.B.); terenzivalentina@gmail.com (V.T.); nicolo.mangini@uniroma1.it (N.M.); dellamonaca.m@gmail.com (M.D.M.); federico.laudoni@gmail.com (F.L.); stefano.dicarlo@uniroma1.it (S.D.C.); valentino.valentini@uniroma1.it (V.V.); 2 Oncological and Reconstructive Maxillo—Facial Surgery Unit, Policlinico Umberto I, Viale del Policlinico 155, 00161 Rome, Italy; andrea-batti@libero.it; 3 Implanto-Prosthetic Unit, Policlinico Umberto I, Viale Regina Elena 287b, 00161 Rome, Italy; 4 Private Practice, Studio Dentistico Ciolfi, via degli Elci 39, 00172 Rome, Italy; alessandro5ciolfi@gmail.com

**Keywords:** head and neck osteosarcoma, surgical treatment, reconstruction, free flap, prosthetic rehabilitation, dental implant, quality of life

## Abstract

Head and Neck osteosarcoma is an uncommon disease. Hitherto, the treatment is surgical resection and survival is influenced by the presence of free margins. However, the dimension of the resection may represent a hurdle for an adequate Quality of Life (QOL). Maxillofacial district is a narrow space where the function, esthetics and patient’s relational skills fit together like the gears of a clock. The functional results depend on the type of reconstruction and prosthetic rehabilitation that are both important to guarantee a good aesthetic result and finally increase the patient’s self-esteem. This study aims to report our experience about head and neck (HN) osteosarcoma focusing the attention on reconstructive and dental-rehabilitative problems. It is a retrospective study all patients were surgically treated in our department. Subjects with histological diagnosis of HN osteosarcoma, treated between 2005 and 2017 were included. The demographic characteristics, surgical treatment, eventually secondary reconstruction and prosthetic rehabilitation, performed in the same department, have been collected. The QOL was assessed through the EORTC QLQ-H&N35 (European Organization for Research and Treatment of Cancer Quality of Life Questionnaire-Head and Neck 35) questionnaire. Fifteen patients were enrolled, eight received a free flap microsurgical reconstruction. Dental rehabilitation was performed in five cases and a mobile prosthesis was always delivered. Eighteen implants were inserted in fibula bones for three patients; highly porous implants were used.

## 1. Introduction

Sarcomas are malignant tumors which can arise from nonepithelial tissue (mesenchymal). As the mesenchymal tissue comprises some different kinds of tissue, from bones to soft tissue and this kind of tumor can occur in any of them, sarcomas are commonly classified according to the tissue from which they originated—bone sarcomas or soft tissue sarcomas. Osteosarcoma, chondrosarcoma (CS) and Ewing sarcoma (EWS) [1,2] are the most common sarcomas of bone. Although head and neck (HN) sarcomas are not very common, as the rate varies from less than 1% of the total head and neck cancers and around 10% of all osteosarcomas [1,2,3,4,5], they represent a significant and distinguished kind of tumors that can lead to challenges in the management and risk for mortality/morbidity [1,6]. The incidence of craniofacial osteosarcoma is around 2 to 3 out of 1 million persons per year [4]. An extensive range of onset, between 10 and 50-year-olds was reported [1,2,7]. Guadagnolo and colleagues noted that the vast majority of HN osteosarcomas arise in either the mandible or maxillary bone [8,9] with 85% of cases involving mandible and maxilla (45% mandible and 40% maxilla) [8,10]. A small number of patients present a history of previous radiotherapy (RT), with consequent osteosarcoma, which was likely radiation-induced [3,11,12,13]. These tumors and the ones arising de novo seem to have similar histopathology, appearance and prognosis. Terenzi et al. reported on 4 patients with a RIS (Radio-Induced Sarcoma) and 3 subjects with an HN osteosarcoma [14]. Fibroblastic, osteoblastic, chondroblastic and teleangectatic are histologic subtypes of osteosarcoma. The great number of HN osteosarcomas are high grade [3,11,12]. Osteosarcomas in the head and neck are typically diagnosed at an older age than long-bone OS (Osteosarcoma) but it is reported they have a slightly better 5-year survival [15]. The pivotal treatment is surgery, with adjuvant RT for those cases with close or positive margins or in case of unfavorable prognostic factors [3]. Recent studies have shown an improvement of OS in patients treated with chemotherapy [16,17,18,19]. At the current state of the art, many authors are investigating research in the field of chemotherapy treatment [20,21,22]. The survival of HNOs (Head and Neck Osteosarcoma) in the published literature ranged from 43% to 63% [1,2,3] and 50.96% 5-year survival rate showed by Chen et al. [16]. A patient with craniofacial osteosarcoma treated by surgery alone has a survival which varies, depending on the authors, between 86%, 75% to 50% as reported in the literature and a relatively good clinical outcome, depending on the extent of surgical resectability [3,23,24]. In the multivariate and univariate survival analysis, the positive resection margin was a most strongly negative prognostic factor for craniofacial osteosarcoma [3,19]. The proximity of the tumor to vital structures, might be a challenge to achieve an ideal “wide” resection margin in HN osteosarcoma without potential considerable functional morbidity [1]. Considering the peculiarity of the anatomical region, most patients undergo major surgical demolition with consequent decrease of quality of life and oral functions, such as speech, mastication swallowing. Functional and esthetic restoration in these patients is a challenging task which must always be subject to optimal treatment. The primary purpose of this study is to report our experience of surgical treatment, reconstructive techniques and dental rehabilitation in patients affected by HN osteosarcoma.

## 2. Results

### 2.1. Sample Characteristic

Eighteen patients diagnosed with osteosarcoma were identified from the department database. Three patients were excluded because the documentation was incomplete. Fifteen met the inclusion criteria and were enrolled, twelve (80%) were de novo OS and three (20%) subjects were RIS (Radio-Induced Sarcoma). The chondroblastic subtype comprised 46.6% of cases (7 patients) and osteoblastic 53.4% of the sample (eight patients). Females represented 73.3% of the sample (patients) and 26.7% were males (four patients). The average age at the time of the diagnosis was 38.53 (+/−18.9 SD). The patients were staged by the AJCC 8th edition classification (The American Joint Committee on Cancer) [25] 3 subjects were stage IA, 11 stage IIA and 1 stage IIB respectively. The average follow-up was 7.46 years (+/−4.59 SD), three patients died during the follow up (20%). Seven patients received only surgery (46.6%) and eight subjects received a multimodality treatment (53.4%). All the characteristics of the sample are shown in Table 1.

### 2.2. Surgical Treatment Details

The mandible was involved in seven cases (46.7% of the sample) and the maxillary bone in eight subjects (53.3% of the sample). All fifteen patients underwent the resection and immediately reconstruction surgery either with a reconstructive plate (three patients) or local flaps (two subjects) or the temporalis muscle flap (five patients), one with obturator and four subjects received an immediate free flap microsurgical reconstruction. One Deep Circumflex Iliac Artery Flap, two Free Fibula Flaps and one Latissimus Dorsi Flap were performed. Four patients were treated in a second surgical time by reconstruction with two Deep Circumflex Iliac Artery Flaps and two Free fibula flaps. The anatomical site, the type of resection and reconstruction are shown in Table 2. Negative margins were obtained in twelve patients (80%). In the three patients with positive resection margins, two needed a second resection and definitive negative free margins were achieved. The last patient had marginal excision, close on the skull base and the patient received protons therapy because it was impossible to obtain an extensive resection. Four (26.6%) local recurrences have been observed and required a second re-excision. Surgical margins free of tumor were found in all four patients who suffered disease recurrence.

### 2.3. Dental-Rehabilitation Details

**Figure 1:**
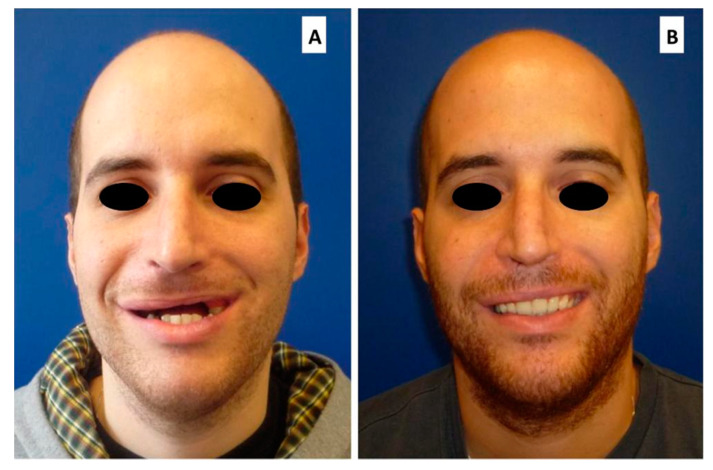
Twenty-five year old male patient affected by osteosarcoma of the premaxilla reconstructed by Free Fibula flap. (**A**) Before prosthetic rehabilitation; (**B**) After final implant supported prosthetic rehabilitation.

**Figure 2:**
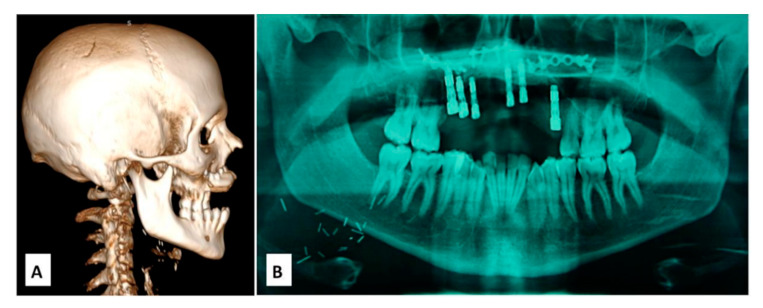
(**A**) CT (Computed Tomography) scan 3D before prosthetic rehabilitation; (B) dental panoramic radiograph after placement of dental implant.

**Figure 3:**
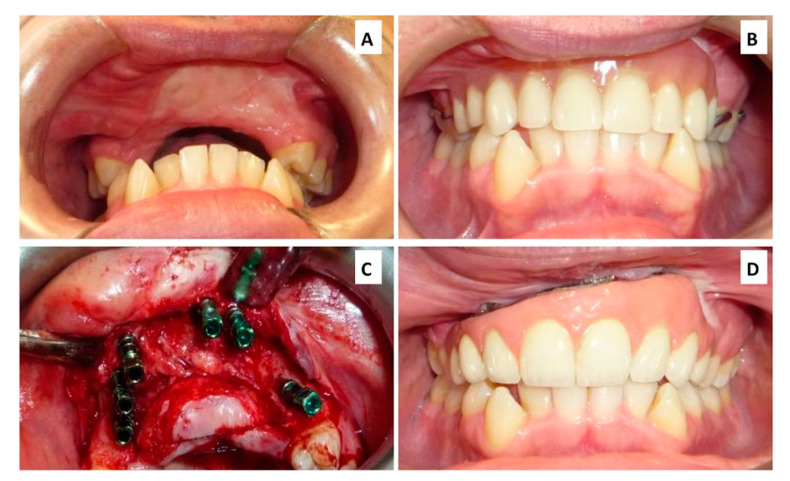
Intraoral view: (**A**) Before prosthetic rehabilitation (**B**) Temporary resin removable prosthesis (custom made). (**C**) Implant placement surgery (**D**) Final implant supported prosthetic rehabilitation.

**Figure 4:**
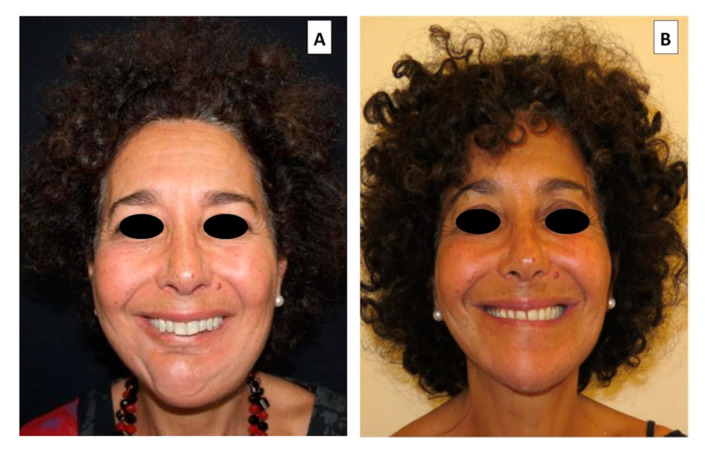
Fifty four year old female patient affected by osteosarcoma of the right mandible and symphysis reconstructed by Free Fibula flap. (**A**) Before prosthetic rehabilitation (**B**) After final implant supported prosthetic rehabilitation.

**Figure 5:**
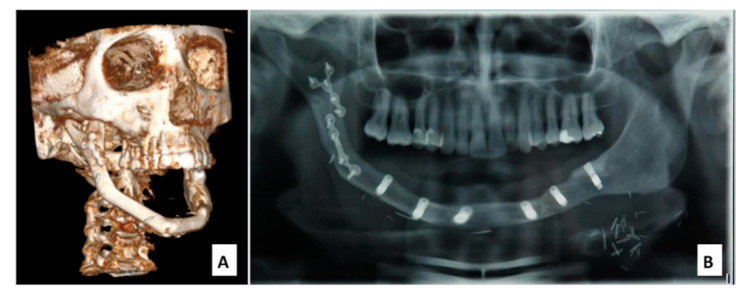
(**A**) CT scan 3D before prosthetic rehabilitation (**B**) OPT after placement of dental implant.

**Figure 6:**
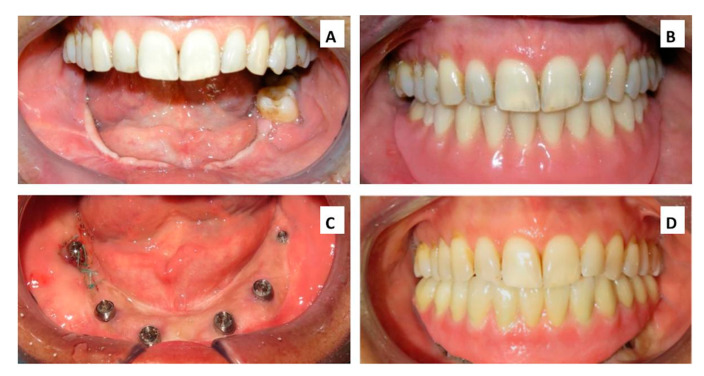
Intraoral view: (**A**) Before prosthetic rehabilitation (**B**) Temporary resin removable prosthesis (custom made) (**C**) Dental implant placed (**D**) Final implant supported prosthetic rehabilitation.

Five patients underwent dental rehabilitation in our Department, two males and three females 52 years old average, three upper maxillae, two in the lower. At first, they were all treated through a common phase aimed at manufacturing a partial or total removable prosthesis to recover the functionality and aesthetics of the stomatognathic system. Subsequently, only three gave their consent to proceed with the fixed rehabilitation phase in our Department. At that point, bone quality and volume were assessed to plan the correct implant design and positioning. Based on the previous temporary mobile prosthesis, a custom-made radiographic guide was manufactured and the CBCT (Cone Beam Computed Tomography) were performed. Then, the custom-made guide was used during the surgery to allow the implants positioning. The implant surgery was performed in local anesthesia and six highly porous implants for each patient were inserted in the site to be rehabilitated. A total amount of 18 implants were inserted in fibula bones for three patients, six for each, two were maxillas (Figure 3) and one mandibular (Figure 6). The subsequent fixed prosthetic rehabilitation was performed differently in the three patients, depending on the location and type of residual defect after maxillofacial surgery. Two explanatory cases of implant rehabilitation are shown in Figure 1, Figure 2, Figure 3, Figure 4, Figure 5 and Figure 6. In one case, after total maxillectomy and free fibula flap reconstruction with persistent oro-antral communication, the prosthesis had to be changed. Then the mobile prosthesis fixed on the titanium bar has been modified to add an additional function, the obturator one, to close the oro-antral communication and restore phonatory and food functionality satisfactorily. An average follow-up of 20 months was evaluated for implant survival. In one case, the reconstructed mandibular patient, three implants had bone reabsorption of 7 mm circumferentially after two years of follow-up. The patient had not been subjected to radiation therapy. The three implants were removed and an immediate collagen bone was grafted in the cavity. Six months later, two short implants (6 mm bone level) and a third (8 mm) were inserted in the same place of the removed ones. The fixed prosthesis was replaced with a mobile one fixed on an implant-supported bar. The average QOL score before prosthetic rehabilitation, obtained from the filling of the EORTC QLQ-H&N35 (European Organization for Research and Treatment of Cancer Quality of Life Questionnaire-Head and Neck 35) questionnaire [26] for the assessment of the quality of life, was 75.8 and the average QOL score after rehabilitation was 62.4. Lower values are linked to an improvement in symptoms. Due to the limited number of samples, it was not possible to evaluate the significance of these values, which however show a positive trend.

## 3. Discussion

### 3.1. Surgical Treatment

HN osteosarcoma has an incidence of 2 to 3 out of 1 million persons per year [4] in the population, it constitutes less than 10% of primary tumors and among craniofacial subsites the mandible and maxilla are frequently involved [23]. The sample consisted of 15 patients, including 3 RIS [14]; the average age was 38.53 (+/−18.9 SD). Hitherto, the treatment is the surgical resection and the clinical outcome and survival are influenced by the presence of negative margins [3]. However, the resection can represent a hurdle for an adequate quality of life and the functional results depend on the type of reconstruction and prosthetic rehabilitation that we can offer the patient. The complex anatomy of the oral cavity, the restricted space and the complexity of the functions it performs make the reconstruction of the maxillomandibular district a challenging situation. It is, essentially, a narrow space where function, esthetics and the patient’s relational skills fit together like the gears of a clock. The maxillary complex is a fundamental structure which is related both to aesthetics and function, supporting the orbital content and the maxillary teeth, dividing the oral and nasal cavities and supplying attachment for mastication and facial expression muscles [27,28]. Mandibular reconstruction needs to restore mastication, speech, swallowing and respiration functions together with dental rehabilitation [29]. Since the face of an individual is their interface with society, deformity of the lower third of the facial contour can create psychological repercussions, which can lead to social distress. The final esthetic result must be the best possible. Psychological distress can drive to isolation and depression [30,31,32]; the reconstruction should limit this consequence. The main problem in the surgical treatment of patients affected by osteosarcoma is to ensure a “wide” resection and negative margins. Resection should be extended to at least 1.5 cm of uninvolved tissue around the mass or an anatomic equivalent, such as periosteum, if allowed by the adjacent vital structures. The risk of recurrence increases below one centimeter [1,33,34]. The age of the patient should also be assessed in surgical planning as we will describe later in the reconstructive choice. The debate is open on the type of reconstruction and timing. Small size lesions may involve reconstruction with local flaps but it is rare in the case of osteosarcoma. Only in 2 patients included in the study, it was possible to perform reconstruction with local flaps. In the case of reconstruction of the upper jaw, the temporalis flap is an excellent surgical option. It is a simple flap to be set up with a shallow failure rate being a pedunculated flap. The esthetic and functional results are variable. Furthermore, depression in the temporal region can be corrected with ancillary techniques (lipofilling and prosthesis). It is also possible to place a mobile prosthesis and rehabilitate the smile. Five patients underwent reconstruction surgery temporalis muscle flap. However, whether the reconstruction takes place in a single surgical time or is delayed, microsurgical reconstruction guarantees the best functional and aesthetic result. It allows to restore the patient’s bone and, in the future, to ensure adequate implant rehabilitation. The choice of the reconstruction type, the donor site and the timing of intervention, above all for osteosarcomas, must be evaluated for each case, whether they involve the mandible or the maxillary bone. It depends on the staging and the presence of negative prognostic factors as well. Although the reconstruction with bone or composite flaps (associated with skin and/or muscle) represents the best surgical choice, dental rehabilitation finalizes the reconstructive result. The choice of the type of flap to use depends on many factors—bone gap extension, the complexity of the defect and the need to reconstruct even soft tissues [35]. For example, the fibula has a longer length and is usually preferred in the reconstruction of long mandible defects involving multiple segments [36]. The DCIA (Deep Circumflex Iliac Artery) flap allows you to use the right amount of bone with the muscle and is generally better for complex defects such as those of the upper jaw. In addition, the quality and thickness of the bone of the iliac crest are higher and, in our experience, it is the best choice to reconstruct even the segmental defects of the mandible [37]. There are also differences in the length of the pedicle and on the morbidity of the donor site [38]. The dilemma of whether to surgically reconstruct or not immediately with free flaps derives from the risk of not obtaining a “wide” resection and therefore having to operate immediately, with the risk of having to sacrifice the flap totally or partially. It also occurred from our experience that the sample 2 patients had an invasion of the margins. One of these had undergone reconstruction with a free fibula flap and one DCIA flap, both needed a second immediate resection of the positive margins and both obtained definitive margins free. Furthermore, when the resections are significant, if possible, it is preferable to temporarily place a reconstructive plate on the jaw or an obturator on the maxilla and wait for the final result of the histological examination. With the certainty of having performed an extensive resection with the confidence of free resections margins, then microvascular surgery can be safer performed. Moreover, in young patients, the attention must be focused on the possible growth of the craniofacial structures over time and therefore the reconstruction with free bone flaps should be deferred at the end of the developmental age [39]. In that way, one of our patients was operated on for the first time at the age of 11. Despite the extensive maxillary resection, the reconstruction was done with the temporalis muscle flap, to program a reconstructive surgery with free flap at the end of growth. However, the patient was satisfied, and she did not want to undergo further reconstructive surgery or implant-prosthetic rehabilitation.

### 3.2. Dental-Rehabilitation

The improvement of oral rehabilitation can be achieved through dental implants that refine upon chewing, language and aesthetics of the oral cavity. The results obtainable are better compared to the mobile prosthesis. [30,40]. The EORTC QLQ-H&N35 questionnaire [26], filled before and after dental rehabilitation, shows average values of QOL improvement after treatment. The small number of the sample does not allow to evaluate the significance of the result, which however points out an improving trend. Said et al. describe the benefits of prosthetic rehabilitation—the use of implant-retained dentures in head and neck cancer patients allowed the most favorable masticatory function [41,42]. Patients with implant-retained prosthesis had better oral rehabilitation than those who did not have on a prosthesis [43,44,45]. Oral function and prosthetic satisfaction were assessed by filling questionnaires to evaluate QOL. The results showed more satisfactory results in patients with implant-retained prosthesis compared to non-implant-retained prosthesis patients, as also assessed in our small sample [46]. Patients who suffer from malignant tumors in the head-and-neck region can be treated with dental implants too. Implant rehabilitation allows proper retention of removable prosthesis with the decrease in the load on soft tissues. It aims to make the life of these patients better. Several factors should be taken into consideration for their influence on the implant survival, above all, when part of the therapy consists in the surgical removal of the tumor. The other essential factors are the surgeon’s experience, bone quality, bone topography and the technical aspects such as implant length, diameter and primary stability. Additional factors’ influence is crucial for implant osseointegration and survival, such as chemotherapy and applied radiation therapy [47]. Soft and hard tissues can be severely affected by radiation or chemoradiotherapy. Total dosage and size of the treatment volume can determine changes [37]. The correct osseointegration and the survival of dental implants can heavily be influenced by side effects such as xerostomia, persistent hyposalivation, changes in bacterial flora, lockjaw, fibrosis of soft tissues, delayed healing and reduced angiogenesis. A prerequisite for maintaining the function of dental implants is the presence of adequate keratinized soft tissue to serve as a barrier at the implant-gingiva junction and this would seem to be the cause of the loss of the implants in our patient. These are the most common side effects able to compromise the success of rehabilitation based on implants. Moreover, poor general health, low level of oral hygiene, smoking and alcohol abuse all reduce implant survival [47,48]. About that, this paper aims to underline the importance of optimal oral rehabilitation for this kind of patients. The consequences of H&N oncologic therapy have a negative influence on QOL and affect all oral functions as chewing and phonation and above all the esthetic of the face. According to the World Health Organization (WHO) criteria, edentulism is a form of physical impairment due to diminished ability to eat and interact socially. The International Classification of Function, Disability and Health (ICF) attempts to place edentulism on equal ground with other noncommunicable diseases [49]. The correct rehabilitation for every single case allows obtaining the best condition both functionally and aesthetically [50]. Psychologically, a right, valid rehabilitation allows the patient to become self-confident again; as a consequence, both social and work-life improves. The optimal rehabilitation has a positive and powerful effect on the patient’s psyche and it allows the start of a non-dysfunctional life. Kende et al. evaluated the QOL using the OHIP (Oral Health Impact Profile) questionnaire and it was also concluded from their results that fixed prosthesis had more positive effects compared to removable partial and complete dentures [51,52]. In this paper, too, the patients’ Quality of Life has been analyzed both before and after the rehabilitation. The data obtained from the EORTC QLQ-H&N35 questionnaire [26], given to each patient to evaluate the effects of the treatment, confirms the improvement in QOL after rehabilitation. The quality of life and speech parameters deteriorated after surgery and improved with use of dental prosthesis (mobile or definitive). The average QOL score after dental-rehabilitation (62.4) decreases compared with the mean QOL score before prosthodontic treatment (75.8), lower values are linked to an improvement in symptoms. It is possible to specifically highlight the reduction in the values that describe—Trouble with social contact, Trouble with social eating, Speech, Saliva and Swallowing. Due to the small number of patients, data obtained from the questionnaire are not statistically relevant; however, they represent an indicative value of the effects of optimal oral rehabilitation.

## 4. Materials and Methods

It is a single-institution retrospective study. Inclusion criteria—(1) patients surgically treated at *Department of Oral and Maxillofacial Sciences, Rome Italy,* (2) between January 2005 and December 2017 with (3) definitive histological diagnosis of osteosarcoma and (4) complete clinical and radiological documentation. All subjects gave their informed consent for inclusion. The study was conducted under the Declaration of Helsinki and the protocol was approved by the Ethics Committee of *“Sapienza” University of Rome*, Italy (N. 4/2020 Prot. n. 0000105). Demographic characteristics were recorded. Patients were staged under the AJCC (8th edition) classification [25]. Duration of follow-up was defined as being the total duration of time from the initial diagnosis to last documented clinical follow-up and was recorded. A descriptive statistic was performed using the SPSS Software. Treatment characteristics included surgical resection, recurrence, type of primary reconstruction and eventually secondary reconstruction where described. Dental rehabilitation has been described for patients who were followed up for dental rehabilitation at *Department of Oral and Maxillofacial Sciences*. EORTC QLQ-H&N35 questionnaire [26] was administered to evaluate the QOL. Implant survival follow-up and complications have been reported.

## 5. Conclusions

HN sarcoma of the bone is a rare disease. Hitherto, the treatment has been surgical resection and survival is influenced by the presence of negative margins [3]. However, the resection can represent a hurdle for an adequate quality of life. The functional results depend on the extension of the resection, type of reconstruction and prosthetic rehabilitation. The quality of life is crucial and adequate implant-rehabilitation improves esthetic and functional results, increasing the patient’s self-esteem. Preoperative evaluation must take into account the staging and the presence of negative prognostic factors that may need adjuvant therapy. In our experience, free bone flaps represent the best method for reconstructing extensive dissections, guaranteeing an excellent functional and esthetic outcome and implant rehabilitation as well. Nevertheless, in order to obtain a definitive histological diagnosis of wide resection margins it is preferable temporarily place a reconstructive plate on the mandible or an obturator on the maxilla and then, in a second time, microvascular surgery can be more safely performed.

## Figures and Tables

**Table 1:** Sample characteristic: simple descriptive statistics performed by SPSS Software.

Sample Size (*n*):	15
Sex:	
Male	4 (26.7%)
Female	11 (73.3%)
Age (mean +/− SD):	38.53 years +/− 18.91
Stage:	
IA	3 (20%)
IIA	11 (73.3%)
IIB	1 (6.7%)
Margins:	
free	12 (80%)
positive	2 (13.3%)
marginal resection	1(6.7%)
Recurrence:	4 (26%)
Treatment:	
Surgery	8 (53.3%)
Multimodality	7 (46.7%)
Follow up (mean +/− SD):	7.46 +/− 4.59

**Table 2:** Surgical treatment and Dental-rehabilitation details.

*N°*	*Site*	*Surgical Procedure*	*Immediate Reconstruction*	*Margins*	*Secondary Reconstruction*	*Dental Rehabilitation*
**1**	Maxilla	maxillectomy Brown II	temporalis flap	free		no
**2**	Maxilla	maxillectomy Brown I	temporalis flap	free		no
**3**	Maxilla	maxillectomy Brown III	temporalis flap	free	DCIA	yes
**4**	Maxilla	maxillectomy Brown III	LD	marginal excision		no
**5**	Maxilla	total maxillectomy	obturator	free	FF	yes (dental implants)
**6**	Maxilla	maxillectomy Brown II	FF	free		yes (dental implants)
**7**	Maxilla	maxillectomy Brown III	temporalis flap	free		no
**8**	Maxilla	maxillectomy Brown II	temporalis flap	free		no
**9**	Mandible	emi-mandibulectomy	DCIA	positive	Resection of the margin and reconstructive plate	no
**10**	Mandible	emi-mandibulectomy	FF	positive	Resection of the margin and reconstructive plate	no
**11**	Mandible	emi-mandibulectomy	reconstructive plate	free	FF	yes (dental implants)
**12**	Mandible	segmental mandibulectomy	reconstructive plate	free	DCIA	yes
**13**	Mandible	segmental mandibulectomy	reconstructive plate	free		no
**14**	Mandible	segmental mandibulectomy	local flap	free		no
**15**	Mandible	segmental mandibulectomy	local flap	free		no

LD: Latissimus Dorsi flap, FF: Free Fibula flap, DCIA: Deep Circumflex Iliac Artery flap.
